# Supplementary material for: A New Cell Line Derived from the Spleen of the Japanese Flounder (Paralichthys olivaceus) and Its Application in Viral Study
Source: Biology (Basel). 2022 Nov 24;11(12):1697. doi: 10.3390/biology11121697 (PMC9774307; doi:10.3390/biology11121697)
Supplement: Supplementary file 1 [file biology-11-01697-s001.zip › biology-2013496-supplementary.pdf]

Supplementary Materials

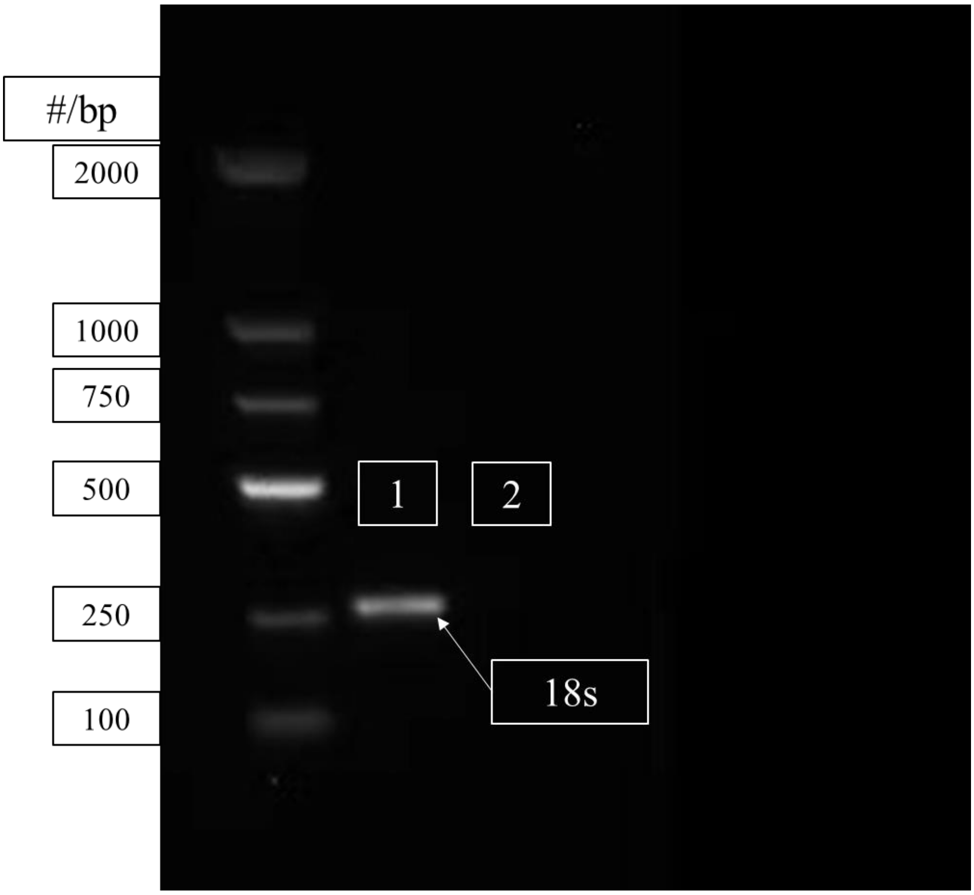

Figure S1. Agarose gel electrophoresis of PCR products from JFSP cells using specific primers for 18s rRNA. M, 2000bp DNA maker; lane 1, 18s; lane 2, Blank. #Weight marker (molecular weight in bp): Sangon Biotech DNA Maker (100~2000 bp), 100 to 2000 bp; catalogue number: B500350.
